# Diameter- and Length-controlled Synthesis of Ultrathin ZnS Nanowires and Their Size-Dependent UV Absorption Properties, Photocatalytical Activities and Band-Edge Energy Levels

**DOI:** 10.3390/nano9020220

**Published:** 2019-02-07

**Authors:** Guanjie Xing, Xiaoli Liu, Simeng Hao, Xiaohong Li, Louzhen Fan, Yunchao Li

**Affiliations:** College of Chemistry, Beijing Normal University, Beijing 100875, China; 201631150029@mail.bnu.edu.cn (G.X.); 15650767721@163.com (X.L.); 201721150019@mail.bnu.edu.cn (S.H.); lxhxiao@bnu.edu.cn (X.L.); lzfan@bnu.edu.cn (L.F.)

**Keywords:** ultrathin semiconductor nanowires, size-controlled synthesis, size-dependent optoelectronic properties

## Abstract

Benefiting from their ultra-small diameters and highly structural anisotropies, ultrathin semiconductor nanowires (USNWs) are well-known for their fascinating physical/chemical properties, as well as their promising applications in various fields. However, until now, it remains a challenge to synthesize high-quality USNWs with well-controlled diameters and lengths, let alone the exploration of their size-dependent properties and applications. To solve such a challenge, we report herein a ligand-induced low-temperature precursor thermolysis route for the controlled preparation of ultrathin ZnS nanowires, which is based on the oriented assembly of the in-situ formed ZnS clusters/tiny particles. Optimized synthetic conditions allowed the synthesis of ZnS nanowires with a diameter down to 1.0 nm and a length approaching 330 nm. The as-prepared ultrathin ZnS nanowires were then intensively examined by morphological, spectroscopic and electrochemical analytical means to explore their size-dependent optical absorption properties, photocatalytic activities and band-edge energy levels, as well as their underlying growth mechanism. Notably, these USNWs, especially for the thinnest nanowires, were identified to possess an excellent performance in both the selective absorption of ultraviolet (UV) light and photocatalytic degradation of dyes, thus enabling them to serve as longpass ultraviolet filters and high-efficiency photocatalysts, respectively. For the ultrathin ZnS nanowires with a diameter of 1.0 nm, it was also interesting to observe that their exciton absorption peak positions were kept almost unchanged during the continuous extension of their lengths, which has not been reported previously.

## 1. Introduction

Ultrathin semiconductor nanowires (USNWs) typically refer to the semiconductor nanowires with diameter well below their exciton Bohr radius, especially for those with an ultra-small diameter (less than 2.0 nm) [1,2]. Compared to regular semiconductor nanowires, USNWs possess stronger size quantum effects, more significant interface effects, and more novel physical and chemical properties due to their ultrathin diameters and highly structural anisotropies, which in turn makes them the ideal building blocks for a variety of applications ranging from nanoscale devices, optical probes, adsorption carriers, to reaction templates. For example, Zhu et al. have observed an interesting ferromagnetic behavior in ultrathin ZnS nanowires of 2.0 nm diameter at room temperature [3]; Niederberger et al. have prepared ultrathin W_18_O_49_ nanowires with a diameter of less than 2.0 nm for serving as the sensing element of a gas sensor for the sensitive detection of H_2_ [4]. Wang et al. have reported ultrathin rare earth hydroxides (GdOOH) nanowires with a diameter of less than 1.0 nm showing a number of appealing polymer-like features [5]; Yu et al. have revealed that ultrathin Te nanowires can be used as a reaction template for creating various one-dimensional (1D) nanostructures due to their high reaction activity [6]; Ozin et al. have demonstrated a gram-scale preparation of ultrathin Bi_2_S_3_ nanowires with a diameter of 1.6 nm, which display an interesting ligand-tuned conductivity [7]. Moreover, benefiting from their unique structural and optical properties, USNWs have also shown a great potential for use in photodetectors [8], light-emitting diodes [9], dye adsorption [10], electrochromic devices [11], and so on.

Despite great efforts being made to synthesize and utilize USNWs so far, it still remains a big challenge to prepare high-quality USNWs on a large scale, especially for the ones with a diameter below 2.0 nm. The challenge lies in the lack of effective means to control the nanowire diameters into a magic size range during their growths, although quite a few methods, like the vapor–liquid–solid (VLS) [12,13], template-assisted deposition (TAD) [14,15], and solution–liquid–solid (SLS) method [16,17] have been developed for preparing regular semiconductor nanowires. This difficult situation stems from the fact that these methods typically require high reaction temperatures (SLS ≥ 270 °C, VLS ≥ 400 °C) and specific-sized catalyst particles (from several nm to tens of nm), or physical temples with specific-sized pores (≥ 20 nm) to accomplish the synthesis. With such limitations, it is particularly difficult to prepare the semiconductor nanowires with a diameter less than 5.0 nm by using these methods, not to mention the nanowires with a diameter below 2.0 nm. Recently, a fascinating nanocrystal growth regime—oriented attachment—has been identified and utilized for the growth of 1D nanostructures [18,19,20,21,22,23], in which the nanowires/nanorods are generated by the oriented assembly of the preformed or in-situ formed nanocrystals along one specific direction. This synthesis strategy shows a special advantage in terms of confining the diameters of 1D nanostructures during their growths, thus rendering it a promising approach for the production of USNWs. For example, Peng et al. [22] have prepared CdSe quantum wires with diameters varying from 1.5 to 6.0 nm at relatively low temperatures by using an alkylamine-induced oriented attachment method. Murray et al. [23] have demonstrated uniform PbSe nanowires with a diameter of 4.0 to 20 nm could be synthesized in solution through a fast oriented attachment route. Liu et al. [2] have synthesized ultrathin ZnS nanowires with a diameter of 1.2 nm by thermolyzing “green” precursors in oleylamine and postulated their growth followed a ligand-controlled oriented attachment mechanism. Zhu et al. [3] have fabricated ZnS ultrathin nanowires with a 2.0 nm diameter by adopting a catalyst-free colloidal synthetic protocol with an oriented attachment assembly regime. It should be noted that although different reaction conditions were utilized in the above cases, they were all believed to have gone through a cluster formation and assembly step sequentially with the help of special ligands before generating the ultrathin nanowires. Such pioneer works stimulate us to find out the suitable conditions for cluster formation and assembly and to directly employ the cluster-based oriented attachment regime for growing high-quality USNWs.

As a kind of important semiconductor material, ZnS nanomaterials are well-known for their excellent optical properties and good biological compatibilities, which have shown numerous applications ranging from ultraviolet (UV) filters and photodetectors to biomolecular detections [24,25,26,27,28,29]. Compared to regular ZnS nanostructures (like nanoparticles, nanorods, and nanospheres), ultrathin ZnS nanowires have a much stronger quantum confinement effect and much more significant interface effect, thus endowing them with a more stronger absorption and photocatalytic activity in the short-wavelength ultraviolet region (especially below 300 nm). Therefore, ultrathin ZnS nanowires are expected to be an ideal candidate material for application in UV filtering and photocatalysis. To solve the challenge in synthesizing high-quality ZnS USNWs, we report herein a ligand-induced low-temperature precursor thermolysis route for the controlled preparation of ultrathin ZnS nanowires via oriented assembly of the in-situ formed ZnS clusters/tiny particles. Optimized synthetic conditions allowed the synthesis of ZnS nanowires with a diameter down to 1.0 nm and a length approaching 330 nm. The as-prepared ultrathin ZnS nanowires were then intensively examined by morphological, spectroscopic, and electrochemical analytical means to explore their size-dependent optical absorption properties, photocatalytic activities, and band-edge energy levels, as well as their underlying growth mechanism. Notably, these USNWs, especially the thinnest nanowires, were identified to possess an excellent performance in both the selective absorption of UV light and photocatalytic degradation of dyes, thus enabling them to serve as longpass ultraviolet filters or high-efficient photocatalysts, respectively. We, therefore, believe this paper is of essential importance in controlled synthesis, in-depth revealing, and better application of such novel ultrathin semiconductor nanowires.

## 2. Materials and Methods

### 2.1. Materials

The zinc dichloride (ZnCl_2_·*x*H_2_O), potassium ethylxanthate, chloroform, methanol and toluene employed in this research are commercially available analytical-grade products and purchased from Beijing Chemical Reagent Ltd Co. of China. Sodium citrate and hexadecylamine (HDA, >90%) were obtained from Sigma-Aldrich (Beijing, China). Octylamine (OA, 99%) was purchased from Aladdin (Shanghai, China). All reagents were used without further purification. Zinc ethylxanthate (Zn(exan)_2_) was synthesized by a method similar to Nair’s [30].

### 2.2. Controlled Synthesis

ZnS USNWs with different diameters were produced by injecting various amounts of Zn(exan)_2_ (dissolved in 4.0 mL OA) into 5.0 g hot HDA solution (150 °C) and were grown at 120 °C for several hours. Specifically, the ZnS USNWs with a diameter of 1.0 nm (model A) were prepared by the injection of 0.4 g Zn(exan)_2_ and reaction for 3 h, while the ZnS USNWs with a diameter of 1.5 nm (model B) and of 2.4 nm (model C) were generated by injection of 0.5 g and 0.625 g Zn(exan)_2_ and a reaction for 3 and 10 h, respectively.

Length control of the ZnS USNWs was mainly achieved by two strategies, namely prolongation of the reaction time or multiple injections of precursor. As for ZnS nanowires (NWs) with different diameters, changing the reaction time was able to tune their lengths to some extent. However, for the ZnS NWs with a diameter of 1.0 nm, multiple injections of Zn(exan)_2_ were necessary to extend their lengths as long as possible. Specifically, additional Zn(exan)_2_ (0.16 g for each time) was replenished into the reaction system three times in order to achieve the longest ZnS nanowires.

### 2.3. Purification and Ligand Exchange

After reaction, the reaction solutions were cooled and precipitated by methanol and were then centrifuged to actualize complete separation. The upper solutions were decanted and the isolated solids were redispersed in chloroform. The above procedure was then repeated several times to fully purify the as-prepared ZnS nanowire products. Finally, the ultrathin ZnS nanowires were redispersed in chloroform or dried under vacuum for further measurements.

To perform ligand exchange, a specific amount of purified ZnS nanowires was added into a flask containing a solution of 0.1 M sodium citrate. The solution was kept at 30–40 °C and continuously stirred for several hours to allow the citrate groups to replace the original alkylamine ligands capping the ZnS nanowires. Finally, the ZnS nanowires that were overcoated with citrate groups were separated by centrifuging and were then dissolved in deionized water for performing photodegradation assays.

### 2.4. UV Filter Preparation and Photocatalytic Test

The UV filters were fabricated by drop-casting a small volume (ca. 0.5 mL) of the chloroform solutions containing the as-prepared ZnS USNWs of various diameters onto different quartz sheets respectively via a polydimethylsiloxane (PDMS) mold with a hollow-carved “BNU” pattern. Afterwards, these sheets were then dried in the vacuum oven to form ZnS nanowire solid films with the “BNU” pattern thereon. Notably, with the above protocol, the “BNU” patterns formed on the quartz sheets were composed exclusively of the ZnS USNWs of various diameters.

The photodegradation assays were performed under 254 UV light (power density 1.35 mW/cm^2^ at a distance of 15 cm, Perfectlight, Beijing, China). Specifically, the as-prepared ZnS nanowire samples (ca. 3.0 mg) after ligand exchange were mixed with 10 ml of Eosin B solution (5.0 × 10^−5^ M) in the quartz bottles and the suspensions were stirred for 1.5 h in the dark to achieve the adsorption–desorption equilibrium before photodegradation testing.

### 2.5. Characterizations

Transmission electron microscopy (TEM) and high-resolution TEM (HRTEM) images were observed by using an FEI Talos 200S high-resolution transmission electron microscope (Born, Czech Republic) operated at 200 kV, accompanied by two electron energy spectrometers (EDS). The specimens for TEM observation were prepared by depositing a drop of the dilute toluene solutions of the purified ZnS nanowire samples on carbon-coated copper grids and drying at room temperature. Powder X-ray diffraction (XRD, Rigaku, Tokyo, Japan) analysis was performed on a Rigaku D/max-2400 diffractometer operated at 40 kV voltage and a 40 mA current with Cuқα radiation (λ= 1.5418 Å). Ultraviolet-visible (UV-vis) spectra were recorded on a UV-2450 spectrophotometer (Kyoto, Japan). Cyclic voltammograms (CV) were measured by a Zahner Zennium electrochemical workstation (Kronach, Germany) with a conventional three-electrode setting and following a previously reported procedure [31,32,33]. Ultraviolet photoelectron spectroscopy (UPS) measurements were carried out on an AXIS ULTRA DLD photoelectron spectrometer (Kronach, Germany) with a base pressure of 3.0 × 10^−8^ Torr and using a He I lamp (hγ=21.22 eV) as the excitation source.

## 3. Results and Discussion

### 3.1. Controlled Synthesis of Ultrathin ZnS Nanowires

**Scheme 1:**
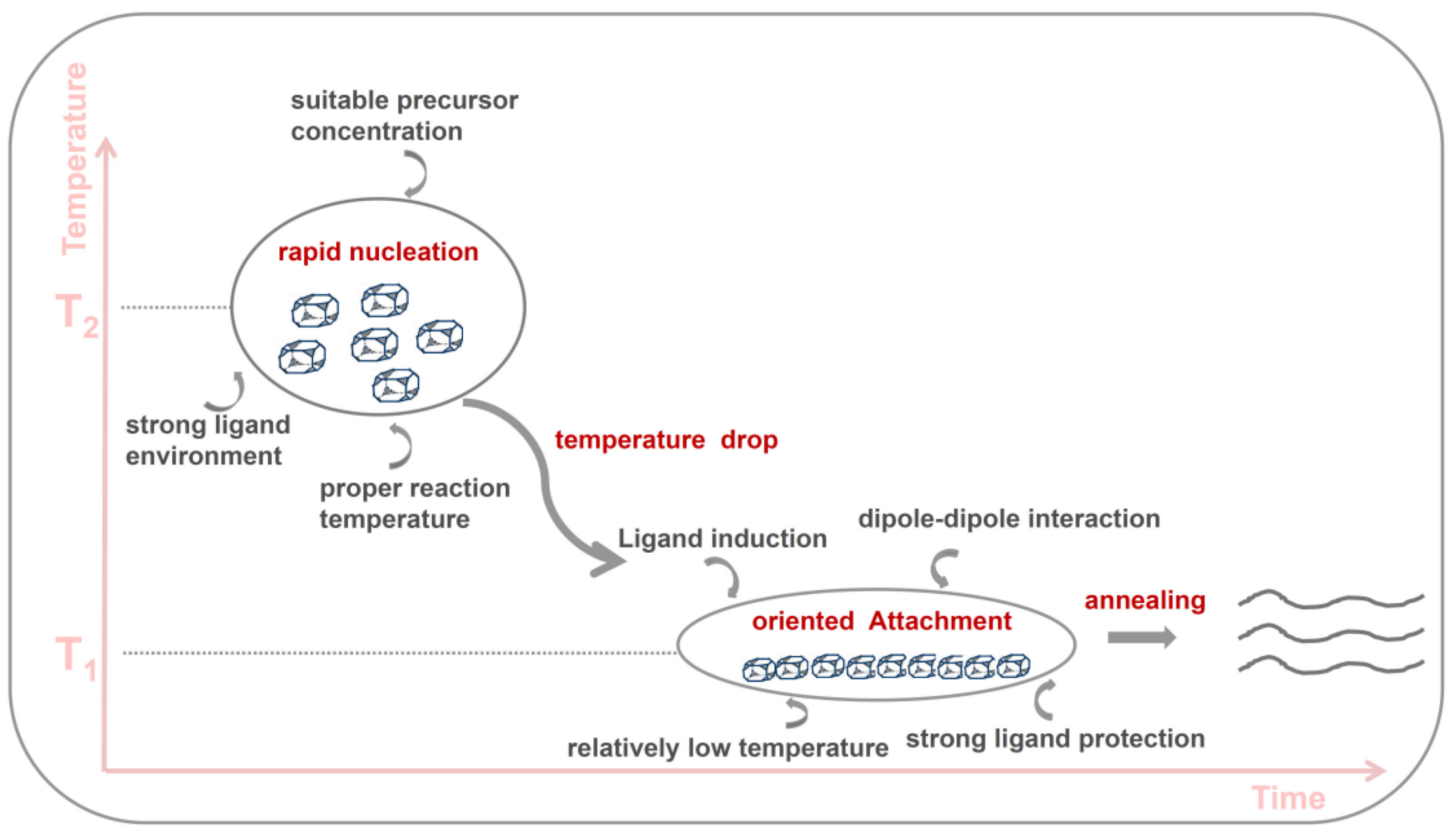
Sketch highlighting the special reaction conditions under which the formation of ultrathin ZnS nanowires through oriented attachment is possible.

**Synthesis Protocol**: As mentioned above, it seems that oriented attachment is a promising approach for the growth of ultrathin nanowires in solution [18,19,20,21,22,23]. However, two prerequisites must be met before such a strategy can be really used to prepare ultrathin nanowires: The presence of stable clusters in the reaction system, and the formation of a special growth environment in favor of cluster-oriented assembly rather than individual growth. As far as cluster formation is concerned, it is generally accepted that the integration of high reaction temperature, high precursor concentration, and strong ligand environment together in a reaction system will facilitate the generation of tiny particles (i.e., clusters) at the nucleation stage [21,34]. However, upon nucleation, such special conditions, particularly, the high reaction temperature and the high precursor concentration, will easily cause the rapid growth of the resulting clusters, so that the clusters cannot retain their original sizes even if they can assemble together though oriented attachment. In this context, it appears that tuning these reaction conditions and the precursor reactivities to a proper level and allowing them to compromise each other may be an effective way to solve the cluster stability issue. As for the confinement of cluster growth during their oriented assembly, from the point of view of crystal growth, it is reasonable to perform such an assembly at a relatively low temperature in the presence of selective binding ligands [34,35,36]. This is because a low reaction temperature typically provides a weak driving force for growing clusters, while ligand binding can selectively passivate specific facets, which are both helpful to restrict the growth of the resulting clusters during their assemblies. It should also be noted that the occurrence of cluster-oriented assembly is still highly dependent on the structural features of the forming clusters and the binding behaviors of the capping ligand molecules, as these two factors have a close relationship with the interparticle dipole–dipole interaction and the highly selective facet fusion [1,4,23,34,37]. Therefore, according to the above consideration, Scheme 1, we designed a low-temperature single-source precursor thermolysis route to prepare ultrathin ZnS nanowires; reactive zinc ethylanthate (Zn(exan)_2_) was chosen as the precursor for its easy decomposition at low temperature; alkylamines of different chain lengths were served as the reaction solvents and the capping ligands simultaneously because of their activation effect and selective binding ability; rapid and a large volume of precursor injection was adopted to intentionally separate the nucleation and growth process by enhancing the temperature difference between them.

**Figure 1:**
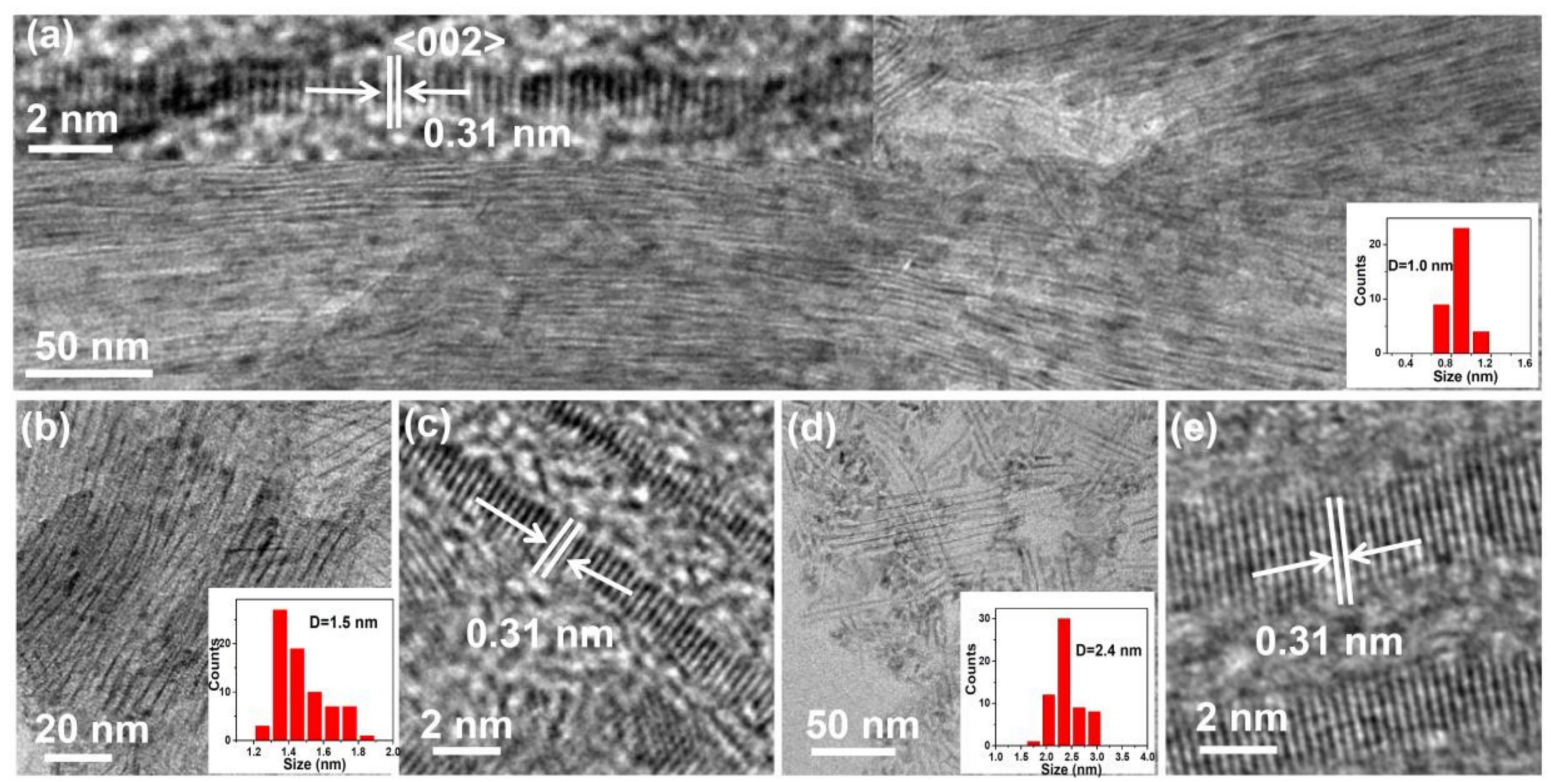
Representative TEM images of ultrathin ZnS nanowires with distinct diameters prepared by thermolysis of different amounts of Zn(exan)_2_ in a hexadecylamine solution (ca. 6.0 mL) at 120 °C for different periods of time: (**a**) 0.4 g of Zn(exan)_2_ and reaction for 3.0 h; (**b**) 0.5 g of Zn(exan)_2_ and reaction for 3.0 h; (**d**) 0.625 g of Zn(exan)_2_ and reaction for 10 h. The insets in (**a**) are its corresponding HRTEM image and the diameter distribution histogram; (**c**) and (**e**) are the HRTEM images corresponding to (**b**) and (**d**), respectively.

**Diameter Control:** Since the diameter is a key parameter to govern the size-dependent optical and electronic properties of semiconductor nanowires, it is thus of particular importance to control such a parameter in the preparation of ultrathin nanowires. Herein, we plan to tune the diameters of ZnS USNWs by modulating the sizes of the originally-formed clusters via changing the precursor concentration at the nucleation stage because the cluster growth is strictly restrained during the assembly process by adopting the ligand-induced low-temperature precursor thermolysis route. As shown in Figure 1, ultrathin ZnS nanowires with diameters of several nm and lengths of several hundred nm were produced by injecting various amounts of Zn(exan)_2_ (dissolved in 4.0 mL OA) into 5.0 g hot HDA solution and growing at 120 °C for several hours. The average diameter of these ZnS nanowires could be tuned from 2.4 nm down to 1.0 nm by reducing the injection amount of Zn(exan)_2_ from 0.625 to 0.4 g. Moreover, the size distribution histograms in Figure 1 and Figure S1 (in the Supporting Information) reveal these nanowires, especially the ones of 1.0 nm diameter, all possess a quite uniform diameter in terms of interwire or intrawire size statistics. HRTEM images together with XRD patterns, see Supporting Information Figure S2, further disclose these nanowires to be single crystals with a wurtzite structure and a strong (002) preferred orientation. It should be noted that the ZnS nanowire with a diameter of 1.0 nm is one of the thinnest ZnS nanowires reported so far, which is quite close to the size of one hexagonal unit cell of wurtzite ZnS [38].

**Figure 2:**
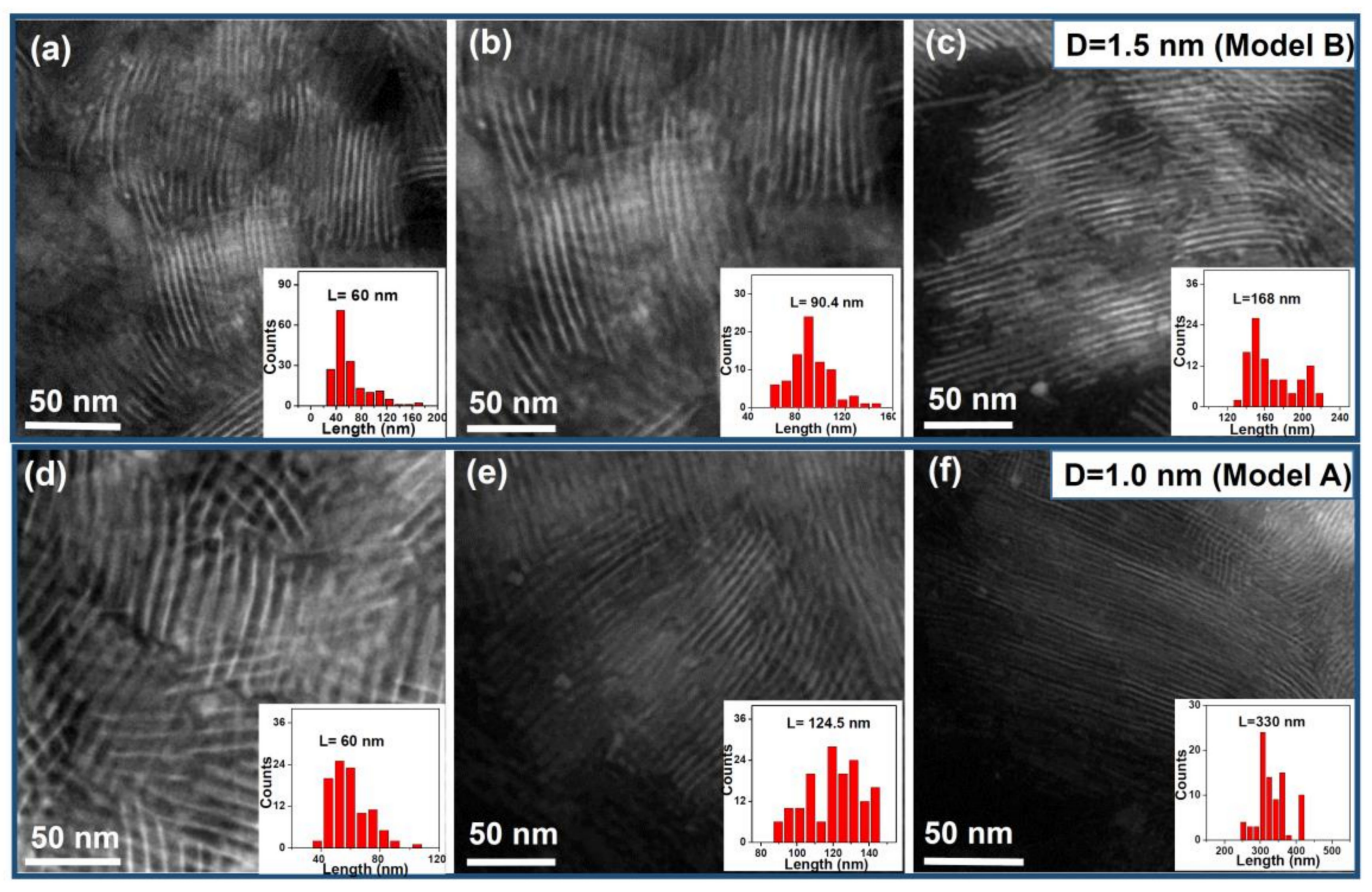
High angle annular dark field–scanning transmission electron microscopy (HAADF-STEM) images to show the length changes of ZnS USNWs of 1.5 nm (model B: **a**–**c**) and 1.0 nm (model A: **d**–**f**) diameter with reaction time: (**a**) reaction for 45 min; (**b**) reaction for 90 min, and (**c**) reaction for 180 min; (**d**) reaction for 25 min after the initial precursor injection, and reaction for another 30 min (**e**) and 90 min (**f**) after the third additional precursor injection. The insets in (**a**–**f**) are their corresponding length distribution histograms.

**Length Control:** Besides the diameter, the length is also closely related to the unique optical and electronic properties of semiconductor nanowires [39,40]. To extend the lengths of the ZnS USNWs without changing their diameters, we mainly adopted two strategies: Moderate prolongation of the reaction time or multiple injection of the precursor. As shown in Figure 2a–c, for the ZnS NWs of 1.5 nm diameter (model B, obtained by injecting 0.5 g Zn(exan)_2_ into 5.0 g hot HDA), their length could be tuned from ca. 60 to 90 nm and then to 168 nm by increasing the reaction time from 45 to 90 min and then to 180 min. It should be noted that the diameters of these ZnS NWs were almost kept the same during such an axial extension process because their radial growths were tightly suppressed. However, by further prolonging the reaction time, the lengths of the ZnS nanowires started to shrink due to Ostwald ripening. Thus, the maximum length to diameter aspect ratio obtained at this condition is ca. 100. In fact, in terms of tuning the nanowire length, multiple injection of the precursor into a reaction system has turned out to be an effective way to extend the lengths of nanowires [41,42]. As shown in Figure 2d–f and Figure S3 in the Supporting Information, for the ZnS nanowires of 1.0 nm diameter (model A, prepared by injection of 0.4 g Zn(exan)_2_ into 5.0 g hot HDA), STEM and HRTEM images both reveal that their lengths could be significantly enhanced with multi-injection of additional precursor into such a reaction system after the formation of primary ZnS nanowires therein; the lengths of the ZnS nanowires were extended from the original 60 to 330 nm after adding extra Zn(exan)_2_ into this system three times (0.16 g Zn(exan)_2_ for each injection). In this case, the maximum length to diameter aspect ratio of the ZnS nanowires could approach 330, thus providing a good platform for investigating the length-dependent chemical/physical properties of USNWs.

### 3.2. Unique Absorption Properties and Applications

**Figure 3:**
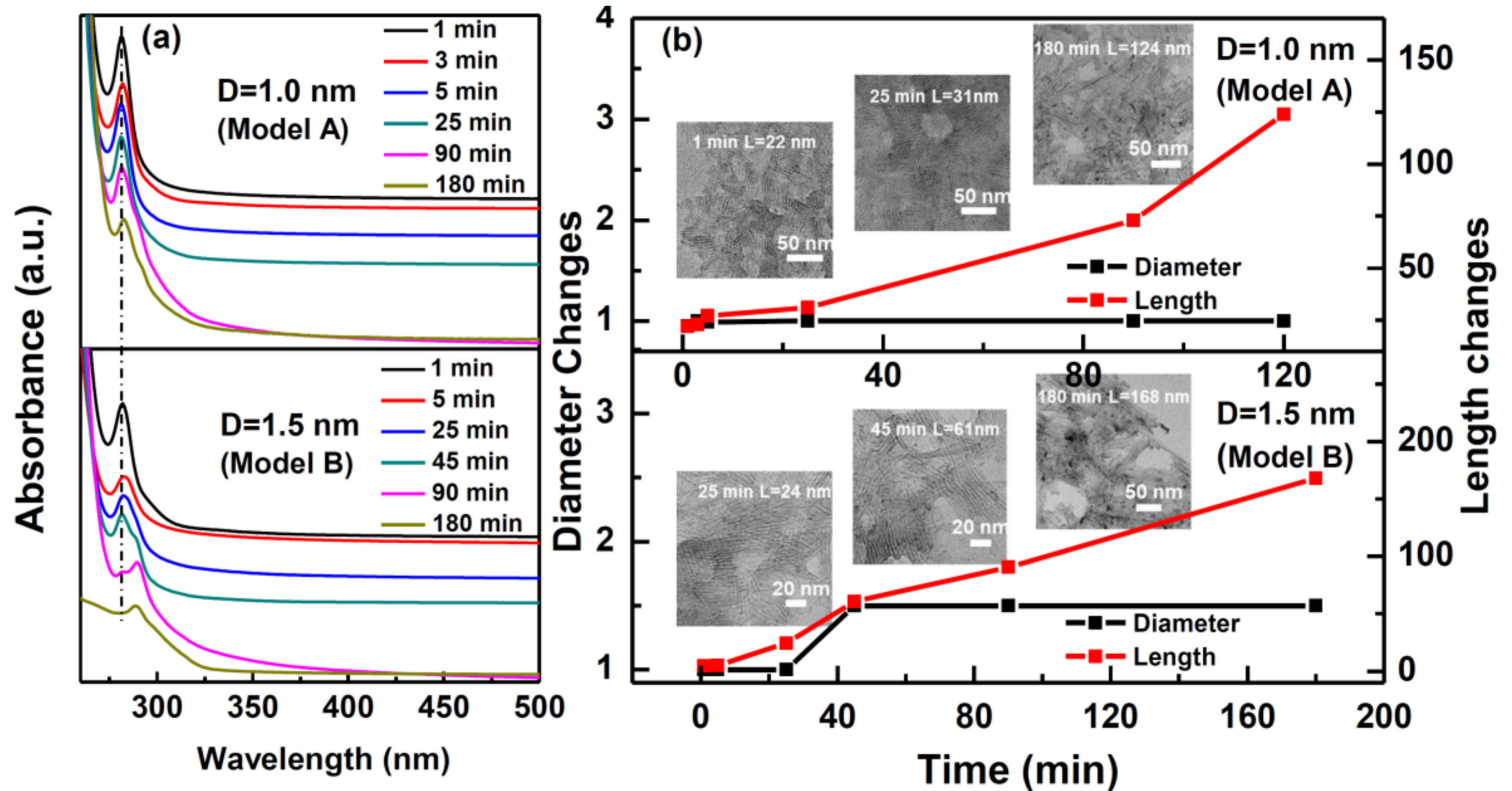
(**a**) Temporal evolution of absorption spectra recording the growth process of the ZnS ultrathin semiconductor nanowires (USNWs) with a diameter of 1.0 nm (model A, left top) and 1.5 nm (model B, left bottom), (**b**) The variation of the length and the diameter of the as-prepared ZnS USNWs with a diameter of 1.0 nm (model A, right top) and 1.5 nm (model B, right bottom) as a function of growth time. The insets in (**b**) are the TEM images of the ZnS nanowires formed at different reaction times.

Absorption spectra of ZnS ultrathin NWs at different growing stages and their length-independent absorption properties: To acquire deep insight into the growth mechanism of the as-prepared ZnS ultrathin NWs and the influence of their lengths on their absorption properties, we took the growth of the ZnS NWs of 1.0 nm (model A) and 1.5 nm (model B) diameter as two examples and systemically compared their absorption spectra recorded at different growing stages. As shown in Figure 3a, in the case of growing the thinnest ZnS NWs (without additional precursor injection), they always display a very sharp absorption peak at 281 nm in the absorption spectra of the samples extracted at different time intervals, indicating the continuous existence of ultra-small products of identical diameter in the entire reaction process [43,44]. According to effective mass model [45],

(1) $$E_{n,gap}=E_{b,gap}+\frac{\pi^{2}h^{2}}{2R^{2}}[\frac{1}{m_{e}^{*}}+\frac{1}{m_{h}^{*}}]-\frac{1.78\;6e^{2}}{\varepsilon R}$$

(where *E_n,gap_* and *E_b,gap_* are the bandgap energy of a semiconductor nanoparticle and its bulk counterpart respectively, *R*, *m*_e_^*^, and *m*_h_^*^ are the radius, electron effective mass, and hole effective mass of the nanoparticle accordingly), the effective sizes of those products are all estimated to be 1.6 nm, which are slightly larger than the values determined by TEM observation. TEM images further reveal the nanorods with the same diameter (1.0 nm, see the size distribution histograms in the Supporting Information Figure S4) but different lengths are actually the main product of all the samples extracted at different reaction intervals, whose lengths were found to extend from several nm to ca. 130 nm by prolonging the reaction time. However, we did observe that many tiny particles (can be named as clusters, ca. 1.0 nm in diameter) were generated in the system at the early stages of the reaction (within the initial 3.0 min) but disappear subsequently as the reaction progresses, see the TEM images and the size distribution histograms in the Supporting Information Figure S5. Therefore, it is highly possible that these nanorods/nanowires were grown from such tiny particles because they both possess an identical diameter. Clearly, the strong and sharp absorption peak located at 281 nm should be exclusively determined by the ultra-small diameters of these ZnS nanorods/nanowires rather than their lengths.

In contrast, when growing the ZnS nanowires with a diameter of 1.5 nm (model B), the absorption spectra recorded at different moments clearly reflect a distinct variation trend. Within the initial 25 min of starting the reaction, only the sharp absorption peak at 281 nm could be detected in the absorption spectra of the samples extracted at different time intervals. As the reaction proceeded, the peak at 292 nm showed up and became dominant at the end, while the peak at 281 nm gradually lost its intensity. The “particle size” corresponding to the 292 nm peak is inferred to be 2.4 nm based on an effective mass model. Thus, the development of a longer wavelength absorption peak along with the disappearance of the shorter wavelength absorption peak might emerge as a result of the smaller-sized product transforming into the larger-sized product. TEM images did confirm the ultrathin nanorods with an average diameter of 1.0 nm and various lengths are the main product generated within the initial 25 min. Noteworthy, some tiny particles (ca. 1.0 nm in diameter, see Supporting Information Figure S6a,d) were also observed to be sporadically distributed among them. However, by further prolonging the reaction time, slightly thicker nanorods were found to emerge as a side product at first (with an average diameter of 1.5 nm, see Supporting Information Figure S6b,e) and then gradually become the dominant product, particularly after 90 min of reaction, see Supplementary Information Figure S6c,f. The peaks at 281 and 292 nm can be attributed to the absorption from the ultrathin nanorods/clusters with a diameter of 1.0 and 1.5 nm, respectively. We, therefore, speculated the final ZnS ultrathin nanowires formed in this case may go through two such intermediate states subsequently, as reported previously prior to essentially extending their lengths [35,43,44,46]. The above results confirm once again that the characteristic absorption peaks associated with the NWs with ultra-small diameters still depend solely on their diameters as expected.

**Figure 4:**
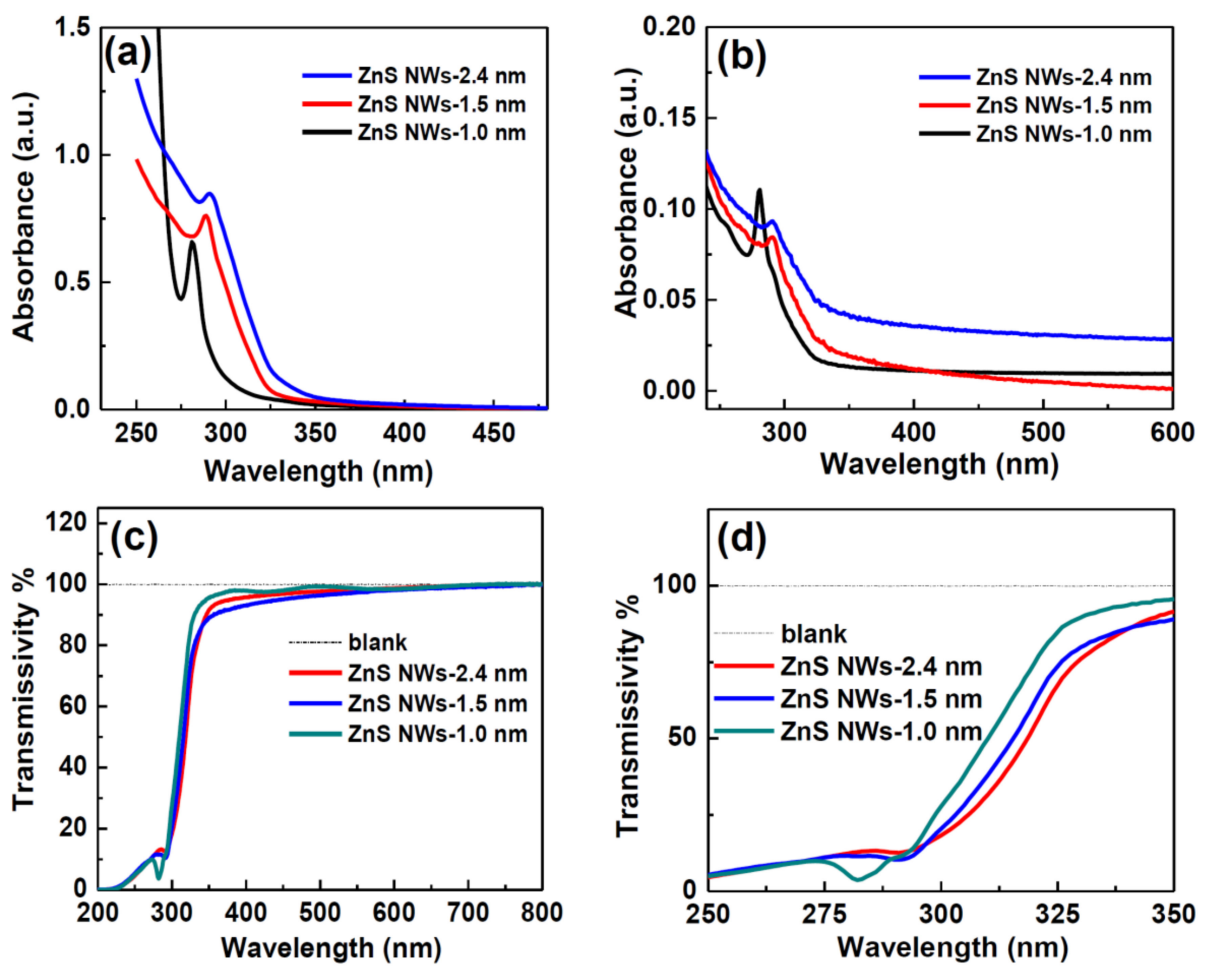
UV-vis absorption spectra of the as-prepared ZnS USNWs of different diameters dispersed in solution (chloroform) (**a**) or spin-coated on quartz sheets (**b**); (**c**) transmission spectra of the three ZnS NW films spin-coated on quartz sheets, (**d**) is a magnified view of the UV region displayed in (**c**).

**Diameter-dependent Optical Absorption Properties and UV Filtering Application**: To investigate how significantly the nanowire diameters affect their optical properties in the case of ultrathin nanowires, we measured and compared the UV/Vis absorption spectra of the as-prepared ultrathin ZnS nanowires of similar lengths but different diameters, respectively. As shown in Figure 4a, these ZnS USNWs all show a sharp and size-dependent excitonic absorption peak in the short-wavelength ultraviolet range (particularly in the UVB region), which is significantly blue-shifted relative to that of ZnS bulk material (344 nm) [47]. The sharp and considerably blue-shifted feature of these absorption peaks indicates these ZnS nanowires all possess a uniform and ultra-small diameter. Meanwhile, the continuous shift of the absorption peak position by decreasing the nanowire diameter also suggests the existence of strong two-dimensional quantum confinement effects in these ZnS nanowires [2,3,48]. According to an effective mass model, their diameters are estimated to be 1.6, 2.4, and 2.9 nm based on the optical absorption edges extracted from their absorption spectra (293, 324, and 331 nm), which are slightly larger than the values measured by TEM.

Similar to the solution-dispersed ultrathin ZnS nanowires, their spin-coating films were also found to display a rather strong and size-dependent absorption peak in the near-ultraviolet region. However, their absorption edges do show a slight red shift (ca. 10 nm) compared to their solution-based counterparts. It is worth mentioning that no obvious absorption could be detected beyond such an absorption region, demonstrating their unique abilities to selectively absorb UV light. Such absorption properties were further confirmed by the transmittivity spectra shown in Figure 4c. It was found that the three as-prepared ZnS nanowire films show near-identical transmittivity behavior in the range below 275 nm (UVC, < 10%) and the one beyond 400 nm (> 90%); however, they do exhibit an inconsistent transmittivity behavior in the range from 290 to 350 nm (particularly in UVB). Such a selective absorption/transmittivity feature endows these ZnS nanowire films with the potential for application as longpass ultraviolet filters with different cut-on wavelengths (i.e., 310, 315, 319 nm, respectively) [49].

**Figure 5:**
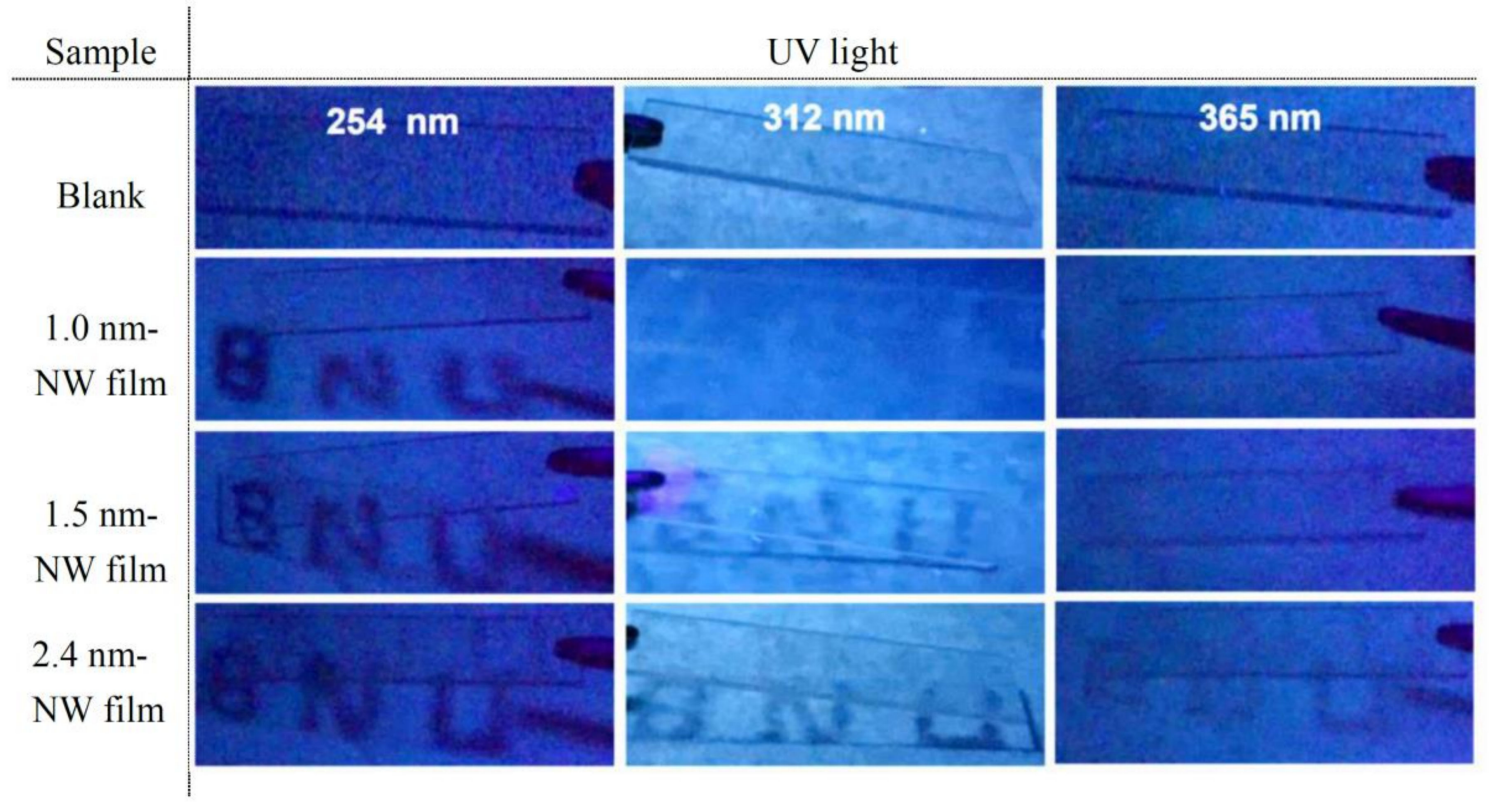
Optical images recording the optical transparencies of four quartz sheets coated with or without (blank) a “BNU” pattern under different ultraviolet (UV) wavelength illumination. The patterns were fabricated by drop-casting a small volume of chloroform solutions containing the as-prepared ZnS USNWs of various diameters onto three quartz sheets via a homemade PDMS mold, respectively.

To test their UV filtering performance, the as-prepared ZnS USNWs were drop-coated on three quartz sheets respectively by using a PDMS mold to form the same pattern of “BNU”, and these sheets were then illuminated under UV lamps of differing emission wavelengths to examine their optical transparencies. As shown in Figure 5, upon exposure to ultraviolet radiation at 254 nm (central emitting wavelength), there appeared a clear “BNU” pattern in a dark color at the locations beneath each quartz sheet (except the blank one), confirming their excellent filtering (absorption) to such a wavelength of UV light. When 312 nm ultraviolet radiation was adopted, only the sheet coated with the thinnest ZnS nanowires (except for the blank one) was observed to allow all the light to pass through (the absence of a BNU pattern), owing to its absorption edge (ca. 310 nm) being shorter than the wavelength of such radiation. In contrast, as 365 nm ultraviolet radiation was utilized, only the sheet coated with the thickest ZnS nanowires did not permit all the light to pass through (the presence of a dim BNU pattern) because its absorption edge (ca. 342 nm) partially overlaps with the wavelength of this UV radiation. Thus, the thin films made from ultrathin ZnS nanowires are ideal longpass UV filters, as they strongly absorb the short-wavelength UV light but allow visible light as well as the long-wavelength UV light to pass through completely. Particularly, their cut-on wavelengths are highly dependent on the diameters of the ZnS nanowires and can be easily tuned in the UVB region.

### 3.3. Size-Dependent Surface Adsorption Properties and Photocatalytic Application

Benefiting from their ultra-small diameters and high length to diameter aspect ratios, the ZnS USNWs are expected to possess large specific surface areas and significant interface effects. To confirm such speculation, we conducted N_2_ adsorption–desorption and dye adsorption assays to examine their surface properties, respectively. According to the adsorption isotherms displayed in the Supporting Information Figure S7a, the Brunauer-Emmett-Teller (BET) specific surface areas of the ZnS NWs with diameters of 1.0, 1.5, and 2.4 nm are estimated to be 143, 98, and 73 m^2^·g^−1^, respectively, which are 2.0–4.0 times higher than that of regular ZnS nanospheres (the control sample, 33.2 m^2^·g^−1^), demonstrating their ultra-high specific surface areas. Upon dispersing into the solutions containing the same concentration of Eosin B, these ZnS NW samples exhibited a remarkable size-dependent adsorption performance in the removal of the dye from solution. With the diameter decreasing from 2.4 to 1.0 nm, the maximum adsorption capacities of these NW samples were found to increase from 40 to 70 mg/g, see Supporting Information Figure S7b–e, all much larger than that of the regular ZnS microspheres (22 mg/g).

**Figure 6:**
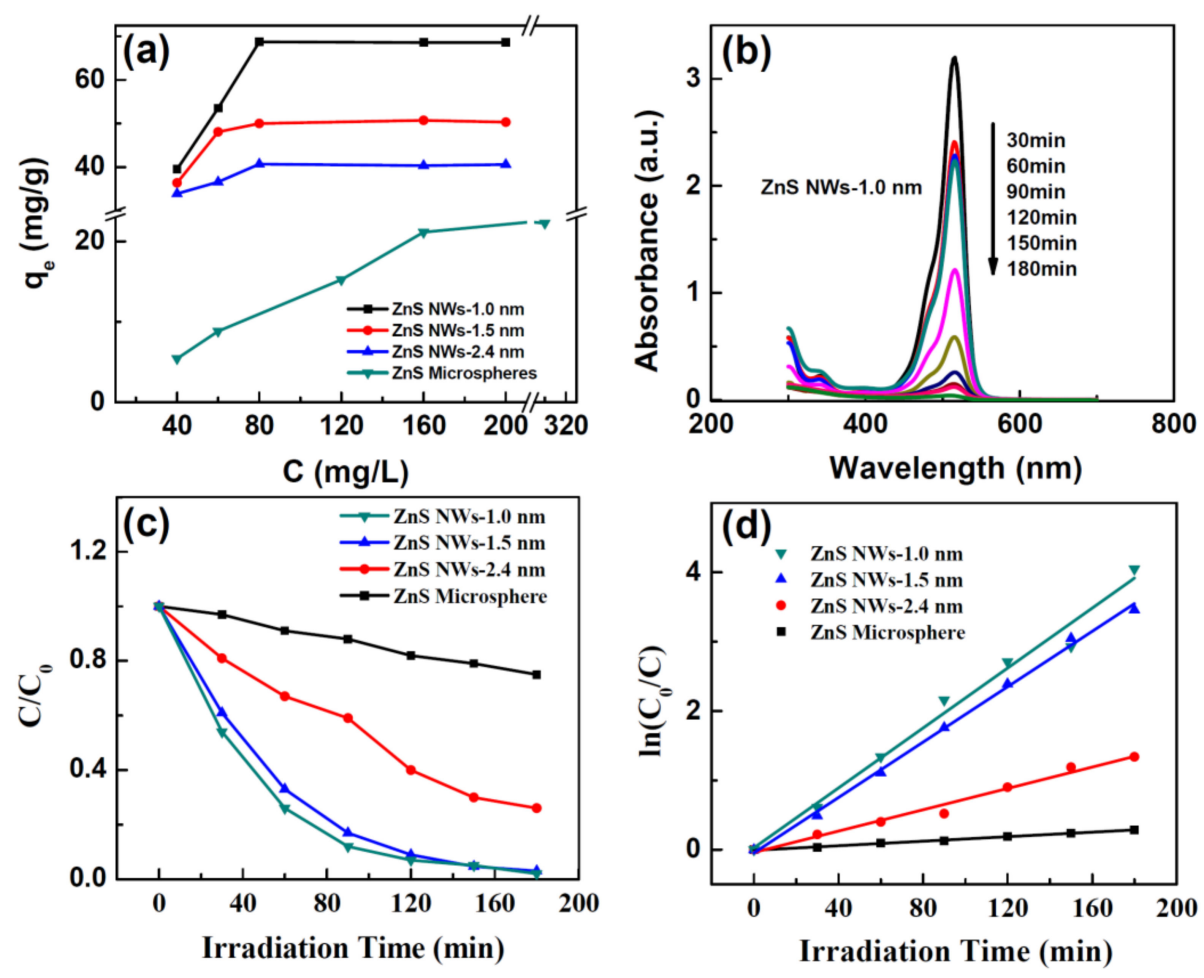
(**a**) The equilibrium adsorption capacity of the as-prepared ZnS USNWs of different diameters towards Eosin B as a function of its concentration; (**b**) temporal evolution of UV-vis absorption spectra of a Eosin B solution (5.0 × 10^−5^ M) in the presence of the ZnS nanowires of 1.0 nm in diameter (3.0 mg) as the catalyst with different exposure periods to UV irradiation; (**c**) normalized concentration of Eosin B in the testing solutions as a function of the illumination time in the presence of various catalysts (e.g., the ZnS USNWs of different diameters or ZnS microspheres); (**d**) plots of ln(*C*_0_/*C*) of the Eosin B solution under test versus irradiation time, displaying the fitting results using the pseudo-first-order reaction model.

Furthermore, the photocatalytic activities of these ZnS USNW samples were evaluated by choosing Eosin B as a target contaminant and monitoring its absorption variation with UV illumination time (Figure 6). As a typical example, Figure 6b shows the temporal evolution of the UV-vis absorption spectrum of an Eosin B solution in the presence of the thinnest ZnS nanowires as the catalyst recorded at a different illumination time (λ = 254 nm). It could be observed that the characteristic absorption peak (at 513 nm) of Eosin B gradually lost its intensity upon prolonging UV exposure and disappeared completely within 180 min, indicating the occurrence of essential degradation of Eosin B under such a condition. In fact, a further comparison reveals the three ZnS NW samples all exhibited a superior catalytic activity towards the photodegradation of Eosin B compared to the control sample (ZnS nanospheres). The photodegradation kinetics of these samples all followed the first-order kinetic model in general [50,51,52]. Among them, it is clear the thinnest ZnS NW sample demonstrated the highest photocatalytic activity; its photodegradation efficiency and rate are nearly four and 13 times higher than that of the ZnS nanospheres, respectively. We think such an excellent photocatalytic performance may stem from the fact that the thinnest ZnS NW sample has the largest specific surface area and the strongest absorption to 254 nm UV light.

### 3.4. Size-Dependent Band-edge Energy Levels

**Figure 7:**
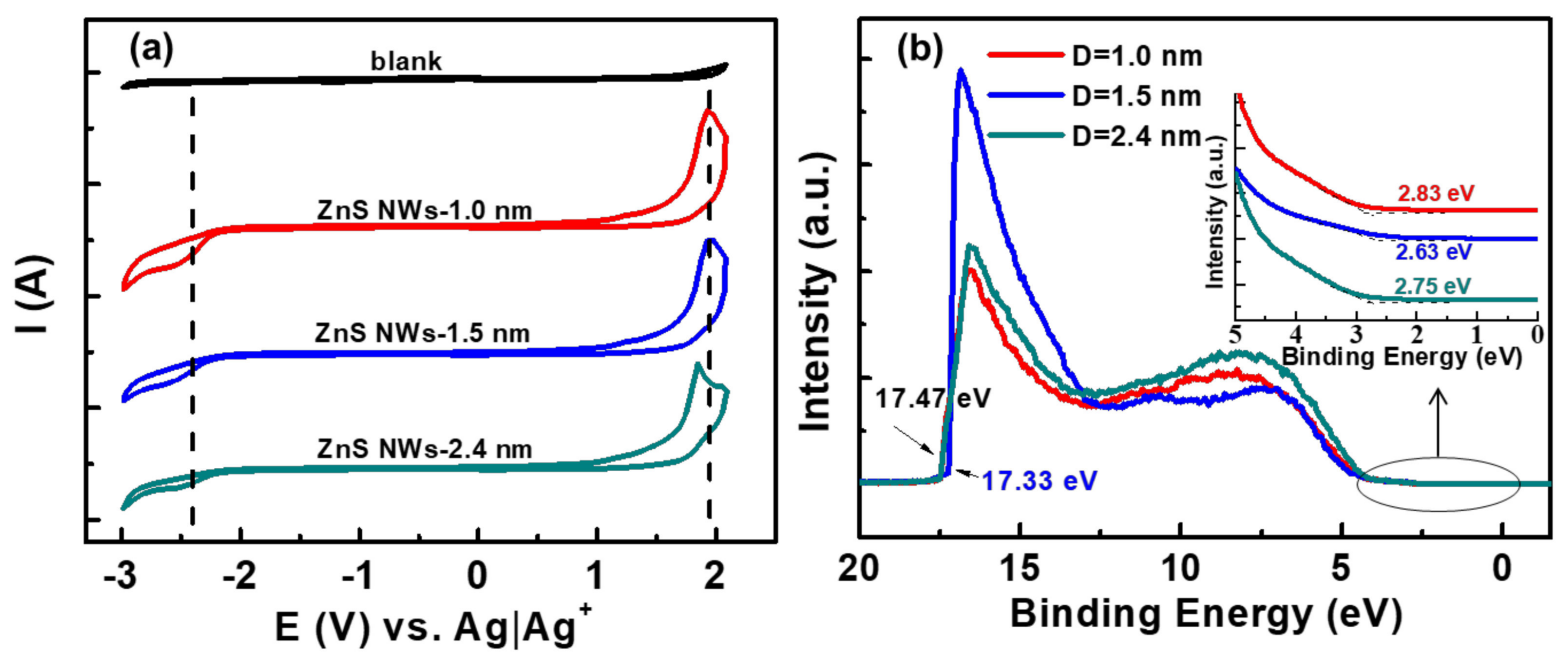
Cyclic voltammograms (**a**) and UPS spectra (**b**) of the as-prepared ZnS USNWs of different diameters. The inset in (**b**) is the magnified view of the low energy edge of the spectra.

To better understand their size-dependent optoelectronic properties, the valence band edge (*E*_vb_) energy level, conduction band edge (*E*_cb_) energy level, and bandgap (*E*_gap_) of the as-prepared ZnS USNWs with different diameters were systemically investigated by cyclic voltammetry (CV) and ultraviolet photoemission spectroscopy (UPS), respectively. It is well-known that the *E*_vb_ and *E*_cb_ energy levels of semiconductor nanocrystals can be respectively estimated from the onset oxidation potential (*E*′_ox_) and the onset reduction potential (*E*′_red_) shown in their cyclic voltammograms [33,34,35]. As shown in Figure 7a, the CV curves of these ZnS nanowire samples all display a pair of well-separated irreversible strong anodic and weak cathodic peaks in the measured potential range, which correspond to the electron loss and gain process occurring at the *E*_vb_ and *E*_cb_ energy level, respectively. However, closer inspection reveals that the onset potential of the anodic and cathodic peaks in each curve are gradually negatively shifted and positively shifted, respectively, upon increasing the nanowire diameter, clearly demonstrating the size confinement effect on both energy levels. Based on previously-established equations [32,33], we could infer that the values of *E*_vb_ and *E*_cb_ energy level of these ZnS NWs are −6.44 and −2.51 eV for ZnS-1.0 nm NWs, −6.41 and −2.50 eV for ZnS-1.5 nm NWs, −6.34 and −2.53 eV for ZnS-2.40 nm NWs, respectively, as listed in Table 1. The *E*_vb_ energy levels of these ZnS USNW were also examined by UPS, see Figure 7b, which were found to be slightly lower than but generally consistent with the values measured by CV, reconfirming the existence of highly size-dependent energy structures in the ZnS USNWs. Besides the energy level of *E*_vb_ and *E*_cb_, the CV measurement can also provide information regarding the *E*_gap_ of the NW samples under investigation. As shown in Table 1, it is clear the *E*_gap_ values of these ZnS USNW samples (except the thinnest one) estimated by CV (i.e., electrochemical *E*_gap_) generally match well with those determined by the optical absorption spectrum (i.e., optical *E*_gap_). However, it is surprising to find that the electrochemical *E*_gap_ of the thinnest ZnS nanowires is marginally smaller than their optical *E*_gap_. We think this inconsistency may be attributed to the presence of strong defect interference in the electrochemical characterization of the thinnest ZnS nanowires.

## 4. Conclusions

In summary, we have presented herein a ligand-induced low-temperature precursor thermolysis route for the controlled preparation of ultrathin ZnS nanowires, which relies on the oriented assembly of the in-situ formed ZnS clusters/tiny particles. With such a route, the diameters and lengths of the ZnS nanowires could be tuned from 1.0 to 2.4 nm and from tens of nm to 330 nm, respectively, by properly varying the precursor amount and reaction time, thereby offering an ideal model system for investigating the diameter- and length-dependent physical/chemical properties of USNWs. Absorption spectrum measurements along with TEM observations revealed the ZnS NWs with a diameter of 1.0 nm were created by the direct assembly of the ZnS clusters/tiny particles of 1.0 nm diameter, while the NWs with a diameter of 1.5 nm were generated in a stepwise fashion, starting from the clusters/tiny particles of 1.0 nm in diameter followed by the assembly of the in-situ formed short nanorods of 1.5 nm in diameter under a higher precursor concentration. The as-prepared ZnS USNWs were identified to possess a remarkable size-dependent UV-light absorption property, which was found to exclusively depend on their diameters rather than their lengths. As a result, their spin-coated films could exhibit an attractive diameter-related optical transmittivity behavior in 290–330 nm, thus enabling them to serve as the longpass ultraviolet filters of different cut-on wavelengths. Moreover, these ZnS NWs also displayed a notable size-dependent performance in the adsorption and photodegradation of Eosin B. Among them, the thinnest ZnS NW sample demonstrated the best performance; its saturated adsorption capacity and photodegradation efficiency were found to be approximately three and four times higher than that of ZnS nanospheres, respectively. Notably, the size-dependent band structures of these ZnS NWs were systemically investigated by CV and UPS respectively, which clearly mapped out the influence of the size confinement effect on the variation of the *E*_vb_ and *E*_cb_ energy level as well as the bandgap. We think the novel synthesis of ZnS USNWs together with their remarkable size-dependent optical properties reported herein will shed new light on the controlled fabrication of high-quality ultrathin nanowires and on the improved application of their fascinating physical and chemical properties in various fields.

## Figures and Tables

**Table 1:** *E*_vb_, *E*_cb_, and *E*_g_ values of the as-prepared ZnS nanowires (NWs) of different diameters measured by CV and UPS.

Samples	UPS	CV			Absorption
	*E*_vb_/eV	*E*_vb_/eV (*E*′_ox_ /V)	*E*_cb_/eV (*E*′_red_/V)	*E*_g_/eV	*E*_g_/eV
D = 1.0 nm	−6.58	−6.44 (1.73)	−2.51(−2.20)	3.93	4.10
D = 1.5 nm	−6.52	−6.41(1.70)	−2.50(−2.21)	3.91	3.82
D = 2.4 nm	−6.50	−6.34 (1.64)	−2.53 (−2.18)	3.81	3.76

## Supplementary Materials

The following are available online at http://www.mdpi.com/2079-4991/9/2/220/s1, Figure S1: Size distribution histograms to show the diameter variation along axial direction taking six individual ZnS ultrathin nanowires with an average diameter of 1.0 nm as examples, Figure S2: XRD patterns (a) and HRTEM images (b) of the as-prepared ZnS USNWs with different diameters, Figure S3: TEM images to show the length changes of ZnS USNWs of 1.5 nm (model B: a–c) and 1.0 nm (model A: d–f) diameter with reaction time: (a) reaction for 45 min, (b) reaction for 90 min and (c) reaction for 180 min; (d) reaction for 25 min after the initial precursor injection, and reaction for another 30 min (e) and 90 min (f) after the third additional precursor injection, Figure S4: Size distribution histograms recording the length variations of the ZnS ultrathin nanorods/nanowires of 1.0 nm diameter with reaction time: (a) 1.0 min; (b) 3.0 min; (c) 5.0 min; (d) 25 min; (e) 90 mins and (f) 120 mins, Figure S5: A representative TEM image of ZnS ultrathin nanorods/nanowires with diameter of 1.0 nm grown for 1.0 min (a) and 120 min (b) after precursor injection, (c) and (d) are the diameter distribution histograms corresponding to (a) and (b) respectively, Figure S6: A representative TEM image of ZnS ultrathin nanorods/nanowires with diameter of 1.5 nm grown for 1.0 min (a), 45 min (b) and (c) 90 min after precursor injection. (d), (e), and (f) are the diameter distribution histograms corresponding to (a), (b), and (c) respectively, Figure S7: (a) Nitrogen adsorption–desorption isotherms of ultrathin ZnS nanowires of different diameter (after ligand exchange). Temporal evolution of UV-vis absorption spectra of a Eosin B solution (5.0 × 10−5 M) in the presence of (b) ZnS nanowires of 1.5 nm diameter, (c) ZnS nanowires of 2.4 nm diameter, and (d) ZnS microspheres (D = 177 nm) as the catalyst, and (e) in absence of any catalyst.
