# Correction: Xing, G. et al. Diameter- and Length-Controlled Synthesis of Ultrathin ZnS Nanowires and Their Size-Dependent UV Absorption Properties, Photocatalytical Activities and Band-Edge Energy Levels. *Nanomaterials* 2019, *9*, 220

**DOI:** 10.3390/nano10061029

**Published:** 2020-05-28

**Authors:** Guanjie Xing, Xiaoli Liu, Simeng Hao, Xiaohong Li, Louzhen Fan, Yunchao Li

**Affiliations:** College of Chemistry, Beijing Normal University, Beijing 100875, China; 201631150029@mail.bnu.edu.cn (G.X.); 15650767721@163.com (X.L.); 201721150019@mail.bnu.edu.cn (S.H.); lxhxiao@bnu.edu.cn (X.L.); lzfan@bnu.edu.cn (L.F.)

The authors wish to make the following corrections to this article [1]:

Figure 2b,d in the original article were incorrect; the correct images are shown below:

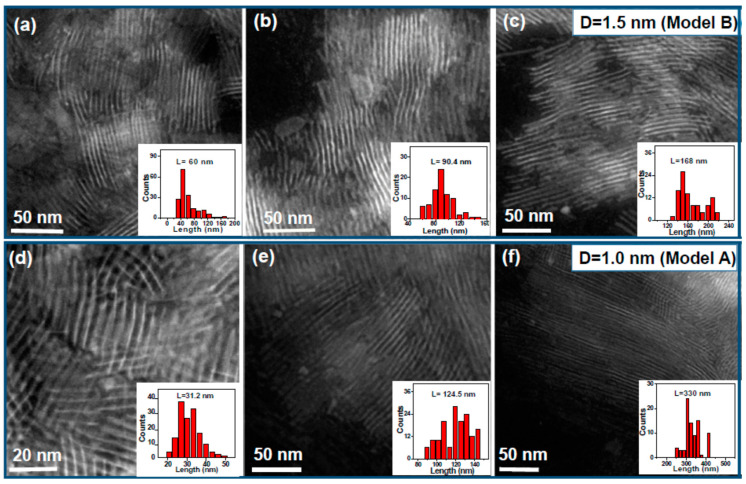



At the end of the “Length Control” paragraph (page 6), the sentence ”the lengths of the ZnS nanowires were extended from the original 60 to 330 nm…” should be “the lengths of the ZnS nanowires were extended from the original ca. 30 to 330 nm…”.

Figure S3d in the Supplementary Materials was incorrect; the correct image is shown below:

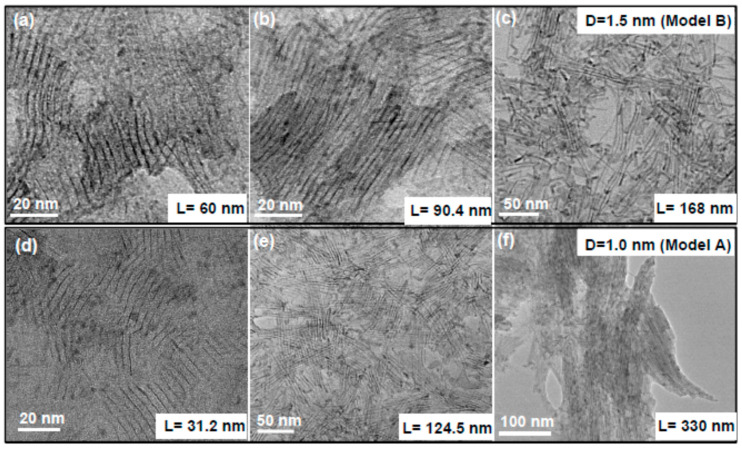



The authors would like to apologize for any inconvenience caused to the readers by these changes.
