# Supplementary material for: Diameter- and Length-controlled Synthesis of Ultrathin ZnS Nanowires and Their Size-Dependent UV Absorption Properties, Photocatalytical Activities and Band-Edge Energy Levels
Source: Nanomaterials (Basel). 2019 Feb 7;9(2):220. doi: 10.3390/nano9020220 (PMC6409554; doi:10.3390/nano9020220)
Supplement: Supplementary file 1 [file nanomaterials-09-00220-s001.pdf]

Supporting Information

for

Diameter- and Length-controlled Synthesis of Ultrathin ZnS Nanowires and Their Size-dependent UV Absorption Properties, Photocatalytical Activities and Band-edge Energy Levels

Guanjie Xing†, Xiaoli Liu†, Simeng Hao, Xiaohong Li, Louzhen Fan, and Yunchao Li *

College of Chemistry, Beijing Normal University, Beijing 100875, China

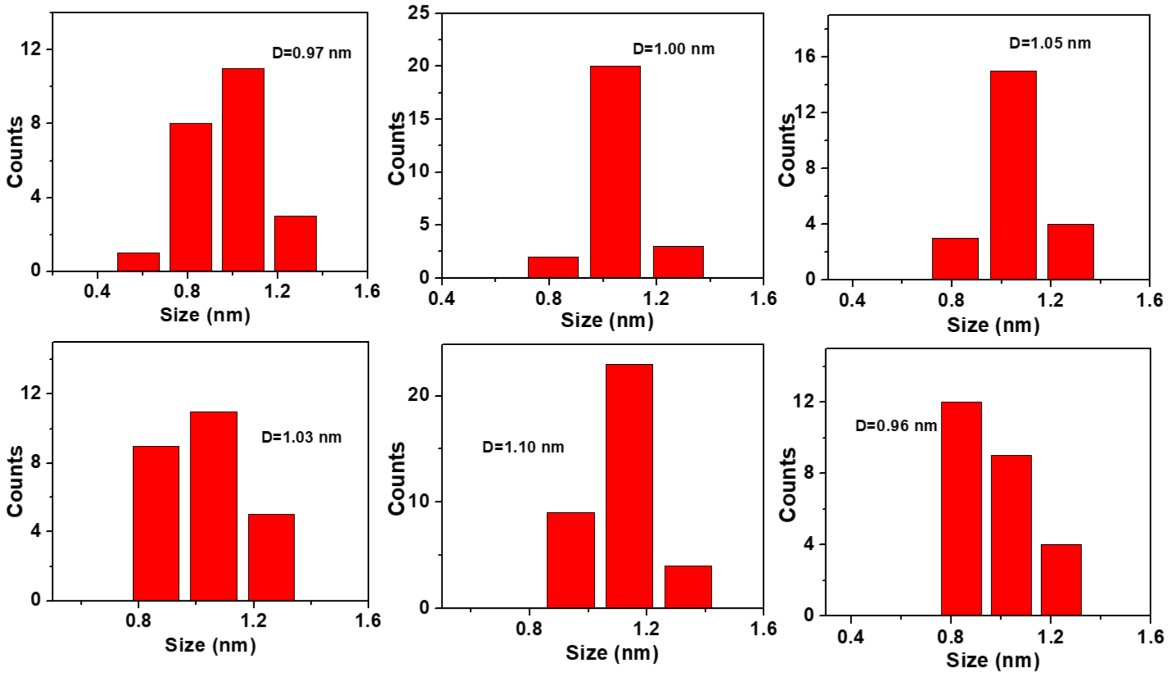

Figure S1. Size distribution histograms to show the diameter variation along axial direction taking six individual ZnS ultrathin nanowires with an average diameter of 1.0 nm as examples.

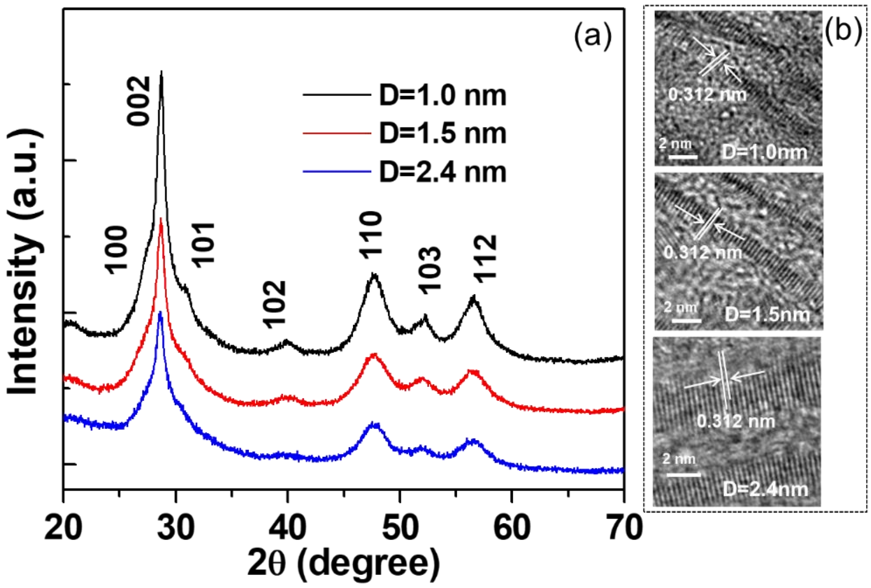

Figure S2. XRD patterns (**a**) and HRTEM images (**b**) of the as-prepared ZnS USNWs with different diameters.

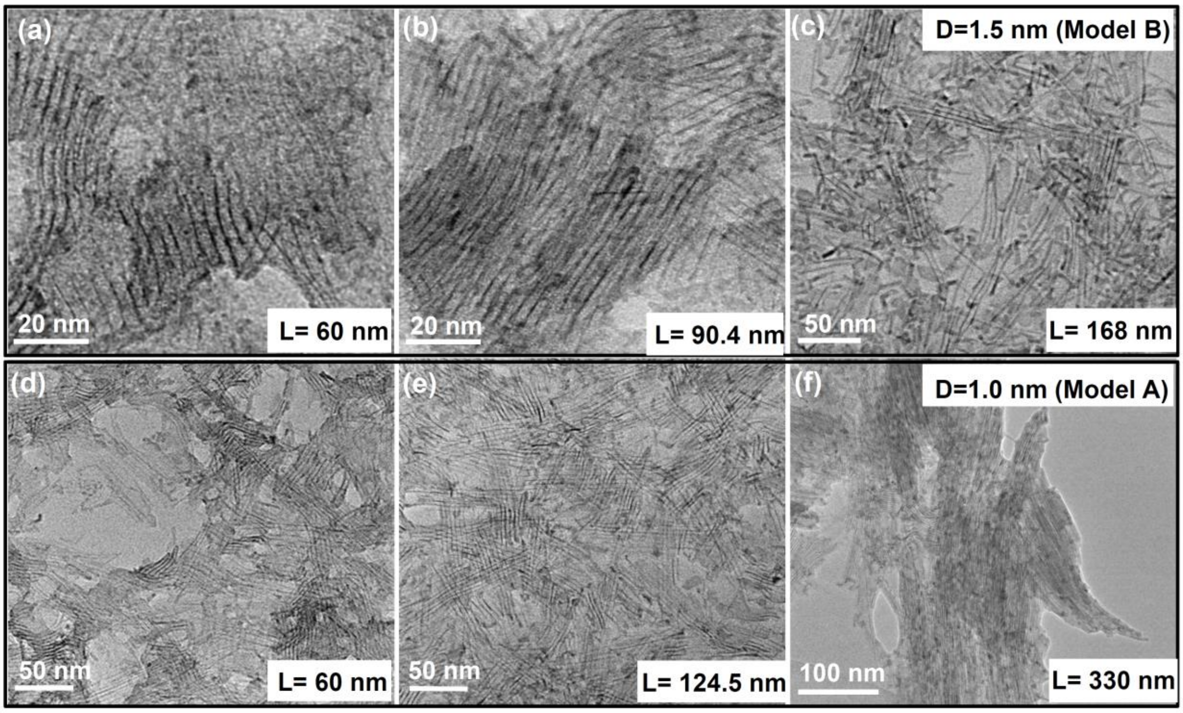

Figure S3. TEM images to show the length changes of ZnS USNWs of 1.5 nm (model B: **a**-**c**) and 1.0 nm (model A: **d**-**f**) diameter with reaction time: (**a**) reaction for 45 min, (**b**) reaction for 90 min, and (**c**) reaction for 180 min; (**d**) reaction for 25 min after the initial precursor injection, and reaction for another 30 min (**e**) and 90 min (**f**) after the third additional precursor injection.

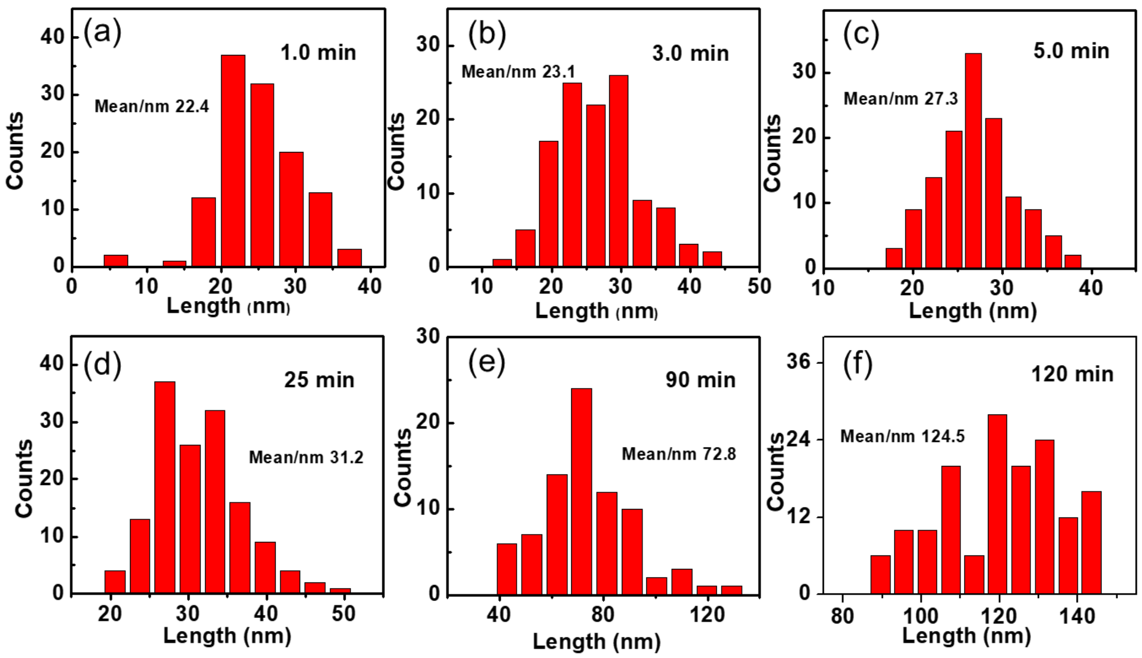

Figure S4. Size distribution histograms recording the length variations of the ZnS ultrathin nanorods/nanowires of 1.0 nm diameter with reaction time: (**a**) 1.0 min; (**b**) 3.0 min; (**c**) 5.0 min; (**d**) 25 min; (**e**) 90 min, and (**f**) 120 min.

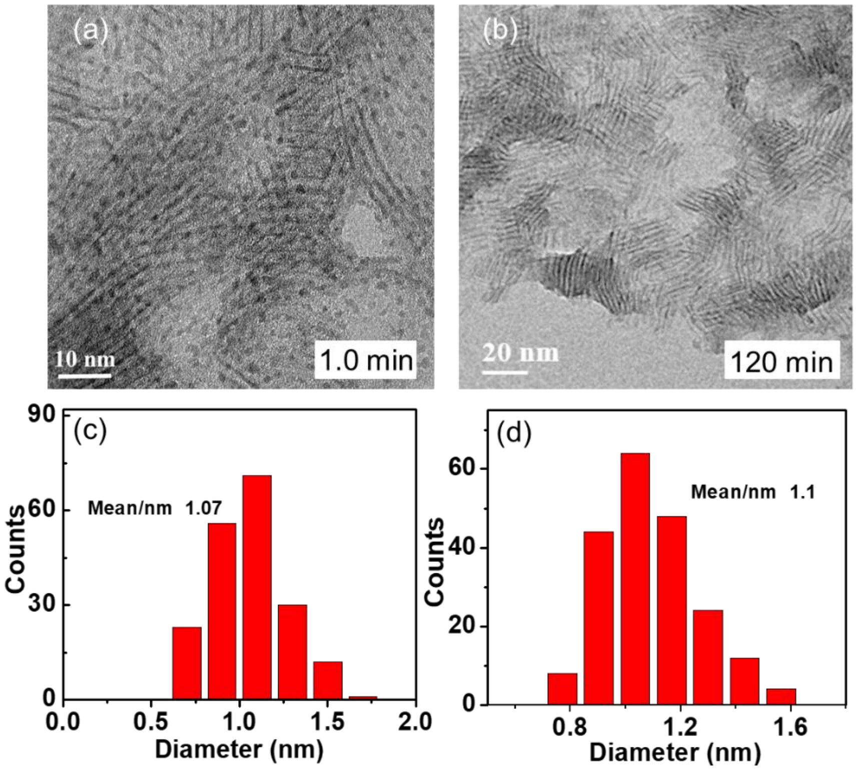

Figure S5. A representative TEM image of ZnS ultrathin nanorods/nanowires with diameter of 1.0 nm grown for 1 min (**a**) and 120 min (**b**) after precursor injection. (**c**) and (**d**) are the diameter distribution histograms corresponding to (**a**) and (**b**), respectively.

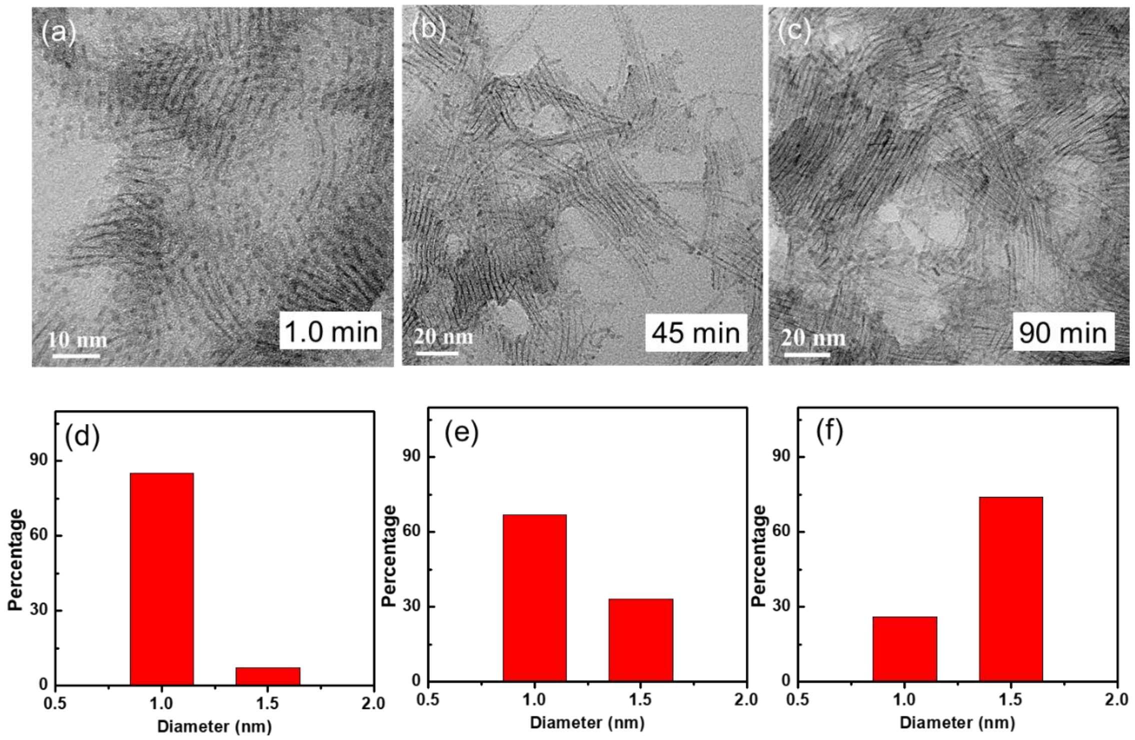

Figure S6. A representative TEM image of ZnS ultrathin nanorods/nanowires with diameter of 1.5 nm grown for 1.0 min (**a**), 45 min (**b**), and (**c**) 90 min after precursor injection. (**d**), (**e**), and (**f**) are the diameter distribution histograms corresponding to (**a**), (**b**), and (**c**), respectively.

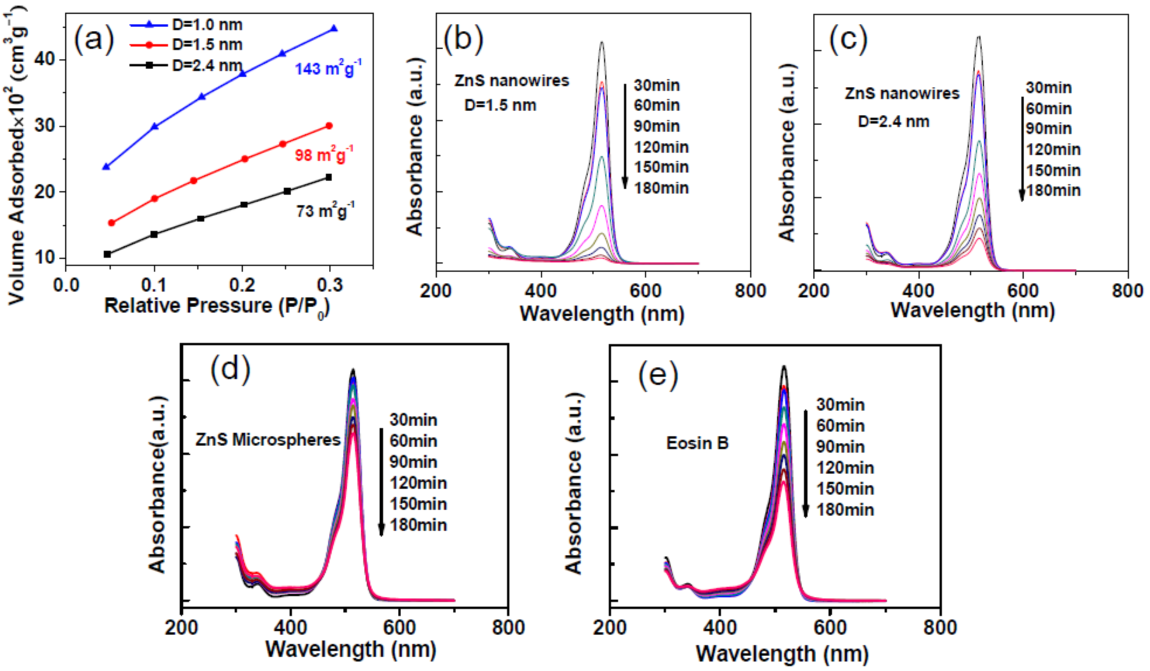

Figure S7. (**a**) Nitrogen adsorption–desorption isotherms of ultrathin ZnS nanowires of different diameter (after ligand exchange). Temporal evolution of UV-vis absorption spectra of a Eosin B solution (5.0×10^{-5} M) in the presence of (**b**) ZnS nanowires of 1.5 nm diameter, (**c**) ZnS nanowires of 2.4 nm diameter, and (**d**) ZnS microspheres (D=177 nm) as the catalyst, and (**e**) in absence of any catalyst.
